# Subjective experience and perception of urban-inland blue spaces in urban parks and individual well-being: evidence from Xi’an, China

**DOI:** 10.3389/fpubh.2025.1621437

**Published:** 2025-10-30

**Authors:** Lixin Wang, Norazmawati Md. Sani, Yuan Wang, Xinrong Xie, Xiaoxiao Zhang, Daoyang Sun, Yanlong Zhang

**Affiliations:** ^1^College of Landscape Architecture and Arts, Northwest A&F University, Xianyang, China; ^2^School of Housing, Building and Planning, Universiti Sains Malaysia, Pulau Pinang, Malaysia

**Keywords:** urban-inland blue spaces, health and well-being, subjective experience, subjective perception, structural equation model

## Abstract

**Introduction:**

The benefits of urban blue spaces in promoting health and well-being have been confirmed by a growing body of research. However, existing relevant studies mostly focus on objective proximity or accessibility and underestimate subjective experience and perception. Therefore, the aim of this study was to investigate the relationship between subjective experience and perception of blue spaces and human health in inland urban public parks.

**Methods:**

To fill this gap, we conducted a field survey of five selected urban-inland public parks with blue spaces in Xi’an, to examine the association of subjective experience of urban-inland blue spaces in parks in terms of their quality, attraction, facility, and management, as well as individual well-being, and to identify the mediating role of subjective perception indicators in terms of social interaction, environmental improvement, recreational activity, and emotional recovery in the relationship.

**Results:**

The study collected 429 valid samples through field surveys and questionnaires, and adopted structural equation modeling to further validate the relationship between subjective experiences and perceptions of urban-inland blue spaces in public parks and individual well-being. The results suggest a potential mediating pathway, and that the quality of urban-inland blue space in parks significantly contributes to well-being outcomes. In addition, indirect pathways have significant associations with outcome variables mainly through subjective perception indicators of pollution reduction and exercise activity.

**Discussion:**

Although this study employed a cross-sectional design and self-reported data, which may impose limitations on causal inference and introduce potential biases. The findings of the study emphasize the importance of the subjective experience and perception of urban-inland blue space in public parks, enriching the evidence on urban-inland blue space planning and public health policymaking.

## 1 Introduction

The beneficial effects of the urban natural environment on human health and well-being have been evidenced by extensive research (1, 2), including improved general health (3–5), enhanced physical activity (6, 7), increased social cohesion (8, 9), reduced individual stress (10, 11), and increased subjective well-being (12–14). In addition, existing evidence supports the theoretical underpinnings of two key frameworks in environmental psychology, including Attention Restoration Theory (ART) (15) and Stress Reduction Theory (SRT) (16). These established theories emphasize the significant restorative effects and psychological benefits of human interaction with the natural environment (16, 17). In current context of rapid urbanization, the importance of the natural environment in improving human health and well-being is of paramount importance (1, 11, 18).

Green and blue spaces, as integral components of the natural environment, play a vital role in enhancing quality of life and promoting physical and mental well-being (19, 20). While the health benefits of green spaces have been extensively documented, research on the potential health-promoting effects of blue spaces has only recently gained momentum and remains an emerging area of study (1). Blue spaces, widely defined as outdoor environments-either natural or manmade-that prominently feature water and are accessible to humans, have received increasing attention from researchers because of their unique landscape characteristics and ecological functions (21–23). Existing evidence suggests that blue spaces, both coastal and freshwater, have more positive impacts on human health, especially mental health (24, 25). For example, previous studies have shown that the accessibility and visibility of blue spaces are significantly associated with the mental health of residents (4, 22, 26, 27).

Based on our review of research on blue spaces and health, existing research focuses on the impact of their objective environmental characteristics on health, such as quantity-based assessment of availability and distance-based assessment of accessibility (4, 24, 25, 28). For example, several studies have adopted the Normalized Difference Water Index (NDWI) (13) or the blue space visibility (27) to assess the availability of blue space at the urban scale. Additionally, the physical distance (Euclidean distance or network distance) or walking time from the residence to the blue space was used to evaluate the accessibility of a blue space (4, 28, 29). In general, standardized data obtained through objective tools or methods is an important method for exploring the relationship between blue spaces and health. However, assessment methods based on objective metrics are generally difficult to accurately describe the specific perceptions and experiences of tourists visiting blue spaces (30–32).

Measures of subjective perception are a self-reported way of assessing a respondent’s specific visit to a blue space, either using questionnaires or face-to-face interviews (29, 33). Subjective environmental perceptions may affect the emotions of people, which in turn influence their behavioral choices (18, 34). For instance, if people positively perceive an environment, they may be more likely to stay and be willing to engage in activities in that environment (18, 35). Despite evidence suggesting that an individual’s subjective perception of the environment may influence their level of mental health to a greater extent than the objective distribution of blue space (18, 31, 34), the available evidence has paid relatively little attention to the individual’s subjective perceptions and experiences. Therefore, in research on blue spaces and health, subjective measurement methods that can capture individuals’ experiences and perceptions in specific environments, as well as their psychological health levels, are worthy of attention from relevant scholars (18, 31).

A study based on previous research on nature and health (2, 17), developed an bespoke model of blue spaces and health that includes three potential pathways: mitigation (harm reduction), instoration (capacity building), and restoration (capacity restoration) (25). Meanwhile, an increasing number of studies have validated the potential mechanisms linking blue spaces to health (11, 13). However, although existing studies validate potential pathway mechanisms, as mentioned above, an often-overlooked dimension of these studies is the subjective experience and perception of blue spaces, which influences the preferences and use of people (18, 30, 31). Notably, existing studies point out that subjective environmental perceptions are associated with health-related behaviors such as physical activity and social contact (11, 18).

Urban public parks contain blue-green spaces and serve the dual functions of improving residents’ quality of life and promoting environmental sustainability (11, 36, 37). Blue spaces in urban parks, especially in inland cities, are among the most accessible blue spaces for residents on a daily basis, and their benefits for improving human mental health deserve greater attention (2, 12, 36). Therefore, based on previous research findings (24, 25, 38), this study focused on the relationship between subjective experiences and perceptions of urban-inland blue spaces in public parks and individual well-being.

**Figure 1:**
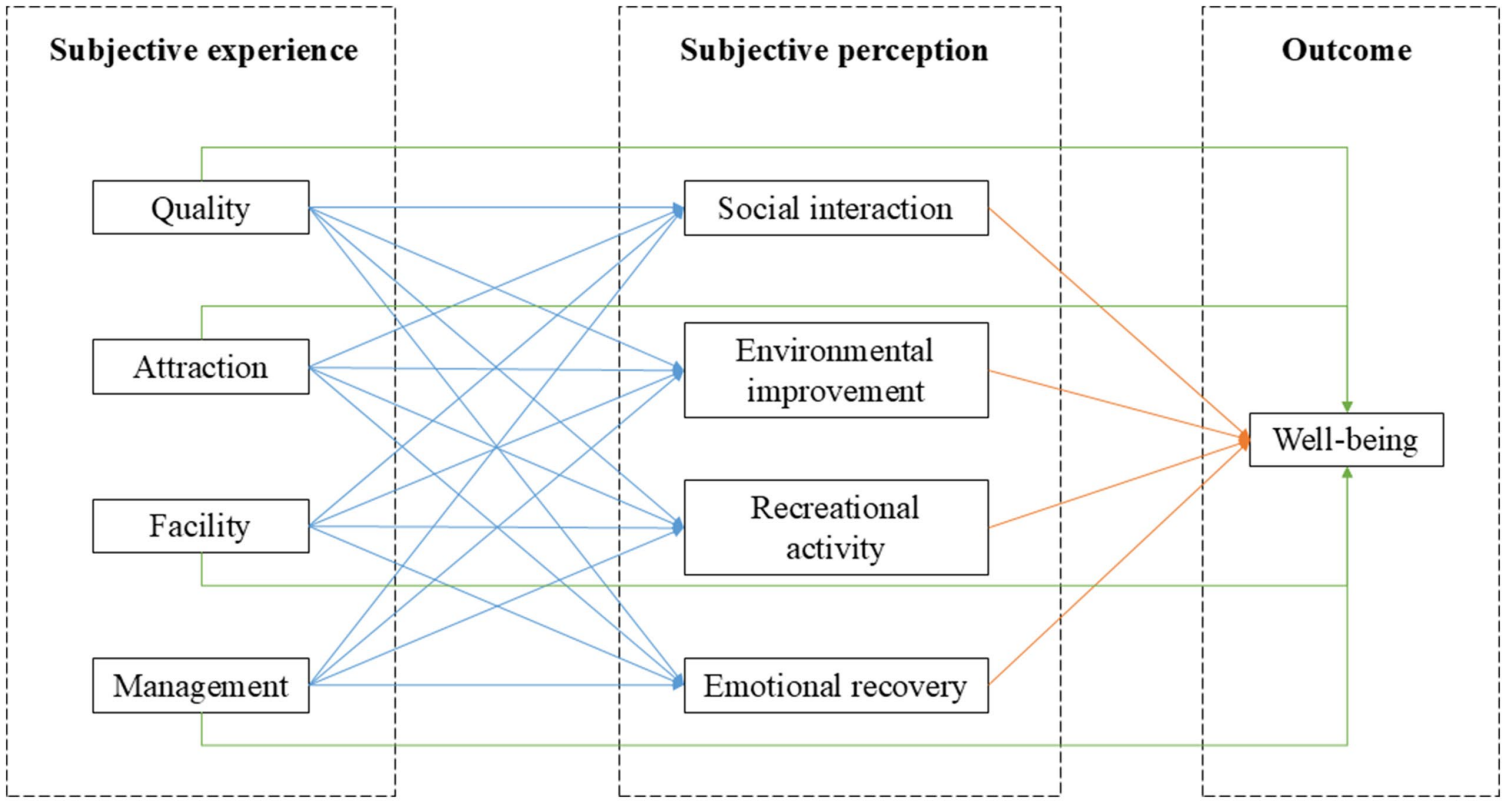
Conceptual framework.

A review of existing studies on blue spaces and health suggested the mechanisms or pathways involved include mitigation, instoration, and restoration (25). Specifically, described as pathways through that blue space can in turn affect health and well-being by harm reduction, capacity building, and capacity restoration, such as reducing environmental harms, promoting physical activity and social connectedness, and reducing anxiety (24, 39). Drawing on these insights, this study constructed a conceptual framework to indicate subjective perception pathways linking the subjective experience of urban-inland blue spaces in public parks to individual well-being (Figure 1).

In this framework, subjective experience is defined as visitors’ direct evaluation of the external attributes of blue spaces, and subjective perception refers to the health-related processes triggered by these experiences. Specifically, considering factors that may influence individual comprehensive experience of blue space, we integrated the subjective experience of the quality, attraction, facility, and management of urban-inland blue space in urban park into the model. In addition, we explored whether or to what extent these indicators of subjective experience influenced the outcome variables through potentially subjective perception pathways (social interaction, environmental improvement, recreational activity, emotional recovery).

Based on individuals’ subjective experience of urban-inland blue space in public parks, this study examined its association with well-being and further analyzed the role of subjective perception in the relationship, with a view to deepen understanding of the mechanism through the urban-inland blue space of public parks play a role in the field of health. Specifically, in this study, blue space refers to water-related features located within public parks in inland urban areas, and the research questions we seek to answer include, first, is there a relationship between the subjective experience of urban-inland blue space in public parks and individual well-being? Second, does subjective perception play a mediating role in this relationship?

## 2 Methodology

### 2.1 Study area

**Figure 2:**
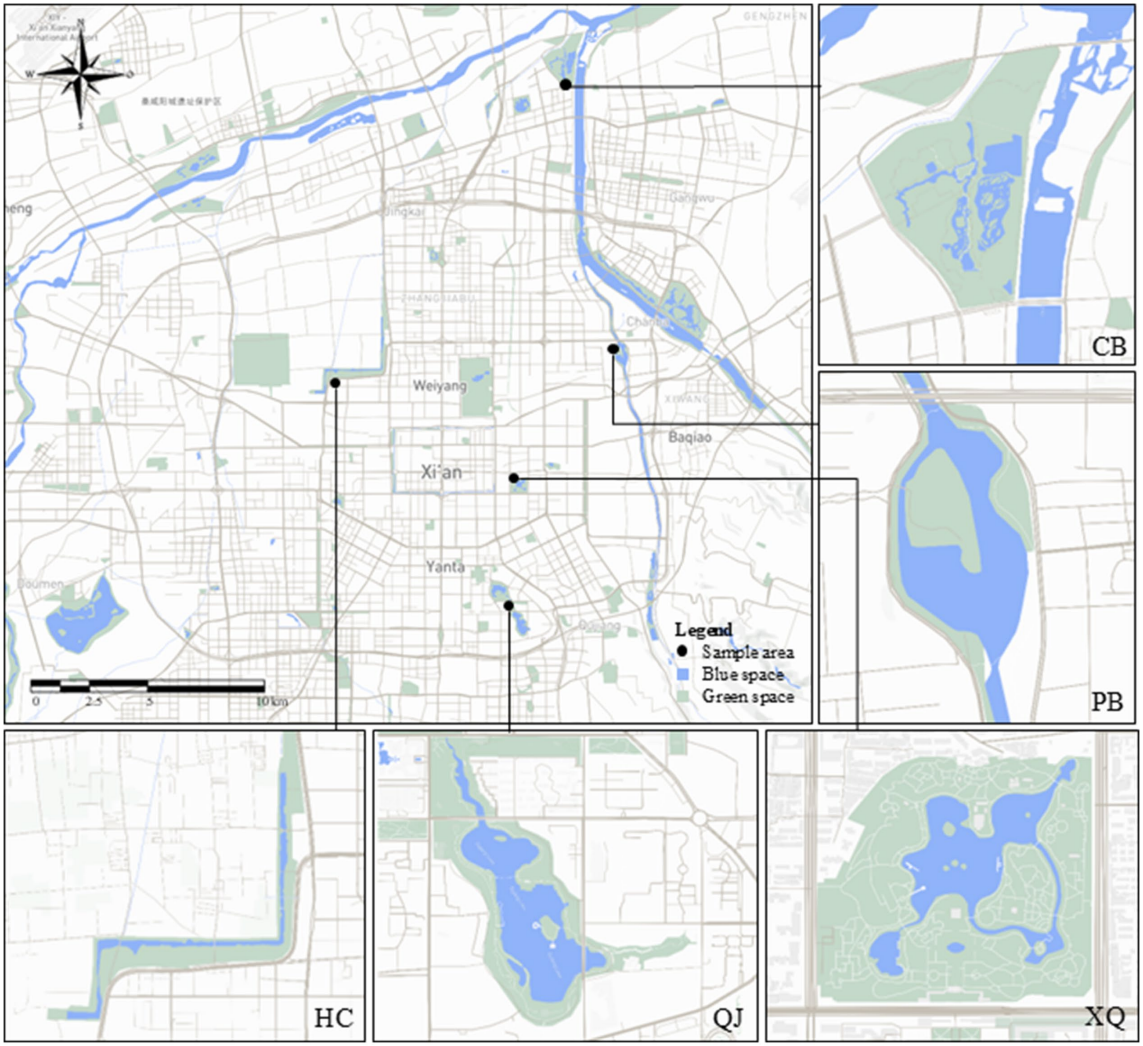
The location of the selected parks. Created using Mapbox.

The study area was selected in Xi’an (107^°^40′–109^°^49′E, 33^°^42′–34^°^45′N), China, a city in the inland northwestern part of the country where valuable water resources make it a unique location to study freshwater blue spaces. Based on field investigations, five urban parks with prominent blue space features in Xi’an were selected as study sites. These parks incorporate visible and physically accessible water elements such as lakes, streams, wetlands, or ponds. All selected parks share common characteristics, including free public access, well-maintained supporting facilities, and convenient transportation accessibility. The five parks included Xingqing Palace Park (XQ), Xi’an Hancheng Lake (HC), Peach Blossom Tan Park (PB), Xi’an Chanba National Wetland Park (PB), and Qujiang Pool Relic Park (QJ). The basic information and specific location of the selected parks are shown in the following Table 1 and Figure 2. Detailed descriptions of the selected parks are provided in Supplementary Table S1.

**Table 1:** Basic information about the selected parks.

Park name	District	Year of completion	Total area (hectares)	Blue space area (hectares)	Blue space rate
XQ^a^	Beilin	1958	52	10	19.2%
HC	Weiyang	2011	289	70	24.2%
PB	Baqiao	2012	40	10	25%
CB	Chanbashengtai	2013	871	200	23%
QJ	Qujiangxinqu	2008	150	30	20%

XQ = Xingqing Palace Park, HC = Xi’an Hancheng Lake, PB = Peach Blossom Tan Park, CB = Xi’an ChanBa National Wetland Park, QJ = Qujiang Pool Relic Park. ^a^This park opened free to the public in 2006, underwent comprehensive renovation in 2020.

### 2.2 Survey data

The study conducted field surveys in five urban parks in Xi’an from April to May 2024, and the questionnaires were collected from adults over the age of 18 who were visiting the selected parks. The questionnaires were collected in the field and the online platform Questionstar was used because it performed well in terms of convenience of data collection and use (https://www.wjx.cn). A convenience sampling method was employed in this study. Visitors in the selected parks were randomly invited to complete the questionnaire, with particular attention paid to ensuring demographic variation in terms of gender and age, and thank you gifts were provided to encourage participation. Ethical approval was provided by the College of Landscape Architecture and Arts, Northwest A&F University.

The required sample size for this study was determined based on G*Power (version 3.1.9.7), using an *a priori* power analysis for linear multiple regression with a medium effect size (f^2^ = 0.15), and a significance level of α = 0.05, power = 0.95 (40), resulting in a minimum required sample size of 153. In addition, following the “10-times rule” recommended by Hair et al. (41) for PLS-SEM, each park was required to include at least 70 participants to ensure the robustness of subgroup analysis. Finally, a total of 429 valid questionnaire was collected across the five selected parks, exceeding the recommended thresholds and ensuring adequate statistical power.

### 2.3 Measurement of subjective experience

Regarding the subjective experience of the urban-inland blue space in public parks, participants were asked to rate the subjective experience of the four dimensions of the blue space being visited, including quality, attraction, facility, and management. First, the subjective experience of the quality (BQ) involved participants’ ratings of the view of the watershed, the color of the water, the design of the waterfront, and the surrounding environment (11, 18). Second, the subjective experience of the attraction (BA) included participants’ ratings of the attraction of the water feature and its landscape resources, and the water feature’s stimulation of interest, curiosity, and creative thinking among respondents (18, 42). Third, the subjective experience of the facility (BF), which investigates participants’ subjective experience of the standardization and adequacy of the facilities around the blue space in terms of pathway, seat, sanitation, and safety amenities (37, 43). Fourth, the subjective experience of the management (BM) of the park’s blue space, which investigated participants’ experience of crowding when visiting the blue space, and their subjective experience of additional fees, behavioral management, and tour guidance (42). The subjective measures employed in this study were derived from previously validated instruments widely used in environmental psychology and public health research, and have demonstrated cultural applicability among Chinese populations (37, 42, 44). The study used a 5-point rating scale to simplify respondents’ reactions and reduce their cognitive stress (1 = ‘strongly disagree’, 5 = ‘strongly agree’). The variables included in each dimension of subjective experience are shown in Table 2.

**Table 2:** Measurement items of subjective experience.

Variable	Item	Question (responses based on 5 points Likert scale)
Quality	BQ1	The waterbody feels open and comfortable.
	BQ2	The water quality is good and its color is pleasant.
	BQ3	The waterfront is well-designed and provides close access to the water.
	BQ4	The landscape of the water feature and its surroundings are harmonized.
Attraction	BA1	The waterscape and surrounding features are attractive.
	BA2	The design of the waterscape captures my interest.
	BA3	The waterscape stimulates my curiosity.
	BA4	The waterscape inspires my creative thinking.
Facility	BF1	The walking paths are accessible and convenient.
	BF2	The rest facilities are adequate and comfortable.
	BF3	The sanitary facilities are appropriate and adequate.
	BF4	The safety facilities are standardized and adequate.
Management	BM1	The level of crowding here is acceptable.
	BM2	The parking fees are reasonable.
	BM3	The behavior of visitor is appropriately managed.
	BM4	The wayfinding system is professional.

### 2.4 Measurement of subjective perception

Following the evidence of blue space and health, factors of subjective perception are conceptualized not as ultimate outcomes, but as intermediary mechanisms through which subjective experience may exert indirect effects on individual well-being. In addition, unlike previous studies that objectively estimated specific pathways, our approach recognizes different understandings of mediating pathways, reflecting the subjective and integral properties of environmental perceptions (18, 31). Therefore, we used participants subjectively assessed perceptions to test potential pathways between blue space and outcome variables. Respondents’ perceptions were assessed through questions on the following four dimensions: promoting social interaction, environmental improvement, recreational activity, and emotional recovery. Response options were presented on a 5-point scale from 1 (strongly disagree) to 5 (strongly agree). The variables included in each dimension of subjective perception are shown in Table 3.

**Table 3:** Measurement items of subjective perception.

Variable	Item	Category	Description
Social interaction	This environment is suitable for social interaction.	Instoration (capacity building)	e.g. promotion of positive outcomes such as improved mood or greater physical activity
Environmental improvement	This environment helps reduce urban environmental pollution.	Mitigation (harm reduction)	e.g. reduction of harm such as urban heat island
Recreational activity	This environment offers a rich variety of recreational activities.	Instoration (capacity building)	e.g. promotion of positive outcomes such as improved mood or greater physical activity
Emotional recovery	This environment is well-suited for leisure and relaxation.	Restoration (capacity restoration)	e.g. recovery from depleted attentional capacity or stress

Category adapted from White et al. (25). “Environment” refers to the blue space within the urban park and its immediate surrounding area.

### 2.5 Assessment of individual well-being

To assess participants’ individual well-being, this study used The World Health Organization Five Well-being Index (WHO-5), a validated instrument used in previous Blue Space and Health studies (21, 36). The WHO-5 consists of five statements that primarily describe the positive emotional states experienced by participants during the past two weeks. The survey consists of a 6-point response scale with options ranging from 0 (no time) to 5 (all the time), and response values are calculated by summing each emotional state score and multiplying by 4 to measure mental health scores on a scale of 0 to 100 (45). Different thresholds have been proposed for the WHO-5 Index, with a score of≥50 generally indicating high well-being, while a score below 50 reflects low well-being (43).

### 2.6 Covariates

We used respondents’ self-reported frequency of visits to green spaces (FG) and blue spaces (FB) in the most recent week as a control variable, assessed on a scale from 1 to 5 (1 = ‘0 days’; 5 = ‘every day’). In addition, respondents made a subjective assessment of the average number of hours per day spent in outdoor spaces in the most recent week (TO), as measured by the following question, ‘What was the average number of hours per day you spent outdoors in the most recent week?’ The response options were categorized into five scales including less than 0.5 h, 0.5 to 1 h, 1 to 3 h, 3–5 h and more than 5 h. According to previous studies, these variables respond to participant’s access to public space as individual factors that may influence the outcome variables of this study (13, 22). As the study focuses on the mechanism linking subjective experience and perception of blue space in urban park with individual well-being outcomes, variables directly related to environmental use behaviors were prioritized for control to reduce model complexity. The description of covariates are shown in Supplementary Table S2.

### 2.7 Analytic method

Structural equation modeling (SEM) allows the direct and indirect effects of independent variables on dependent variables to be measured through different mediators to examine the relationships between key variables and the underlying mechanisms behind them (46). Therefore, we developed a key model for exploring the relationship between blue space in parks and individual well-being based on existing theory and evidence. In this study, we chose to use Partial Least Squares Structural Equation Modeling (PLS-SEM) with Smart PLS, and the prediction-oriented approach of PLS-SEM was applied to this study. Empirical evidence from prior research has consistently demonstrated the methodological appropriateness of PLS-SEM for investigating complex structural models in exploratory research contexts, primarily due to its superior statistical power in detecting and validating theoretical relationships (47). With limited sample sizes, PLS-SEM remains robust to the analysis of non-normal data (31, 47).

Additionally, prior to conducting the structural equation modeling (PLS-SEM), a measurement model assessment was conducted to ensure the reliability and validity of the constructs. Key indicators included Cronbach’s alpha (acceptable if ≥ 0.70), which measures internal consistency; composite reliability (CR) (acceptable if ≥ 0.70), which evaluates the overall reliability of latent constructs; and average variance extracted (AVE) (acceptable if ≥ 0.50), which assesses convergent validity (31, 47, 48). These preliminary assessments provided a robust foundation for the subsequent structural model analysis. Furthermore, the standardized root mean square residual (SRMR) was employed as a model fit index, with values below 0.08 indicating an acceptable model fit. In alignment with established methodological protocols, we implemented a consistent PLS-SEM bootstrap procedure with 5,000 random subsamples to ensure robust parameter estimation and reliable statistical inference (31).

## 3 Result

### 3.1 Descriptive analysis

For this study, 429 participants in total were sampled, including 84 from XQ Park, 72 from HC Park, 95 from PB Park, 97 from CB Park and 81 from QJ Park. Approximately two-fifths (39.9%) of the sample were between the ages of 40 and 49 years old, and the sample of older adults over the age of 60 years accounted for nearly one-tenth (9.8%) of the total sample. In addition, 45.9% of the participants were female, which is comparable to the national average (48.76% of the Chinese female population in 2020). Based on the average, participants reported visiting blue-green spaces on approximately two to three days in the recent week and spent an average of around one to three hours per day outdoors. Overall, the average personal subjective well-being WHO-5 score of the respondents was 78.19 (SD 13.04), which generally indicating high well-being (scores≥50) (43). The descriptive analysis of the key variables is shown in Table 4. Subjective well-being differences across selected parks are provided in Supplementary Tables S3, S4.

**Table 4:** Descriptive statistics of key variables.

Variables	Frequency/mean	Percentage/Standard deviation
Park name		
XQ	84	19.6%
HC	72	16.8%
PB	95	22.1%
CB	97	22.6%
QJ	81	18.9%
Gender		
Male	232	54.1%
Female	197	45.9%
Age		
18–29	54	12.6%
30–39	89	20.7%
40–49	171	39.9%
50–59	73	17%
≥60	42	9.8%
FG	2.85	0.98
FB	2.87	0.99
TO	2.91	0.96
Well-being (0–100)	78.19	13.04

XQ = Xingqing Palace Park, HC = Xi’an Hancheng Lake, PB = Peach Blossom Tan Park, CB = Xi’an ChanBa National Wetland Park, QJ = Qujiang Pool Relic Park. FG, FB, and TO (see Section 2.6 for details) were used as covariates. Categorical variables (e.g., park name, gender, age) were summarized using frequency and percentage, while continuous variables (e.g., FG, FB, TO, Well-being) were described using mean and standard deviation.

### 3.2 Statistical analysis

Table 5 illustrates the indicator loadings and reliabilities of the different dimensions of participants’ subjective experience of blue spaces in selected parks. The results of our model validation analysis showed good internal consistency for all four dimensions of subjective experience. The factor loadings and Cronbach’s alpha values for quality, attraction, facility, and management of the subjective experience of the park’s blue space exceeded the widely recognized threshold point of 0.7, suggesting a good level of reliability (47). The composite reliability (CR) value also passed the recommended benchmark of 0.7, further supporting the internal consistency of the measurement model. In addition, the average variance extracted (AVE) were all above the minimum threshold of 0.5, confirming adequate convergent validity. The variance inflation factors (VIF) for all factors were below the threshold of 2, suggesting the absence of multicollinearity (31, 47).

**Table 5:** Properties of subjective experience measurement.

Variables	Items	Factor loading	Cronbach’s alpha	CR	AVE
Quality	BQ1	0.837	0.797	0.805	0.623
	BQ2	0.786			
	BQ3	0.815			
	BQ4	0.713			
Attraction	BA1	0.785	0.743	0.749	0.564
	BA2	0.759			
	BA3	0.727			
	BA4	0.731			
Facility	BF1	0.767	0.767	0.771	0.589
	BF2	0.739			
	BF3	0.754			
	BF4	0.807			
Management	BM1	0.806	0.780	0.791	0.603
	BM2	0.818			
	BM3	0.732			
	BM4	0.745			

Descriptions of the “Items” are provided in Table 2.

Regarding the relationship between individual well-being and the associated variables, the findings are summarized as follows: First, within the subjective experience dimension of blue space in urban parks, including q quality (BQ), attraction (BA), facility (BF) and management (BM), only BQ demonstrated a significant and positive direct effect on well-being (*β* = 0.439, *p* < 0.001). In contrast, the direct effects of BA, BF, and BM on well-being were weak and statistically non-significant. Second, in terms of the subjective perception dimension, which includes social interaction (SI), environmental improvement (EI), recreational activity (RA), and emotional recovery (ER), both EI (*β* = 0.403, *p* < 0.001) and RA (*β* = 0.136, *p* = 0.013) exhibited significant and positive associations with well-being. However, the effects of SI and ER were minimal and did not reach statistical significance. Third, the control variables, namely green space usage frequency (FG), blue space usage frequency (FB), and outdoor duration (TO) (see Section 2.6), showed weak and non-significant relationships with well-being. Detailed results are presented in Table 6.

**Table 6:** Total pathway effects and significant indirect pathway effects.

Pathway	Effect	*T*	*P*	95% CI
BQ → Social interaction	**−0.163**	**2.470**	**0.014**	**(−0.294, −0.035)**
BQ → Environmental improvement	**0.447**	**5.446**	**<0.001**	**(0.289, 0.612)**
BQ → Recreational activity	0.060	0.799	0.424	(−0.090, 0.203)
BQ → Emotional recovery	0.053	0.837	0.403	(−0.069, 0.176)
BQ → Well-being	**0.439**	**5.718**	**<0.001**	**(0.297, 0.598)**
BA → Social interaction	**0.194**	**2.789**	**0.005**	**(0.064, 0.338)**
BA → Environmental improvement	−0.154	1.483	0.138	(−0.354, 0.053)
BA → Recreational activity	**0.368**	**4.701**	**<0.001**	**(0.218, 0.524)**
BA → Emotional recovery	**0.209**	**2.661**	**0.008**	**(0.063, 0.369)**
BA → Well-being	0.080	0.767	0.443	(−0.124, 0.282)
BF → Social interaction	**0.370**	**4.330**	**<0.001**	**(0.199, 0.536)**
BF → Environmental improvement	−0.070	0.778	0.437	(−0.246, 0.107)
BF → Recreational activity	**0.181**	**2.232**	**0.026**	**(0.016, 0.336)**
BF → Emotional recovery	**0.240**	**3.185**	**0.001**	**(0.089, 0.386)**
BF → Well-being	0.007	0.077	0.939	(−0.170, 0.175)
BM → Social interaction	**0.288**	**3.488**	**<0.001**	**(0.125, 0.445)**
BM → Environmental improvement	**−0.242**	**2.460**	**0.014**	**(−0.438, −0.054)**
BM → Recreational activity	0.095	1.215	0.224	(−0.049, 0.252)
BM → Emotional recovery	**0.284**	**3.993**	**<0.001**	**(0.145, 0.421)**
BM → Well-being	0.061	0.685	0.494	(−0.109, 0.234)
Social interaction → Wellbeing	−0.062	1.142	0.254	(−0.170, 0.044)
Environmental improvement → Well-being	**0.403**	**10.676**	**<0.001**	**(0.327, 0.475)**
Recreational activity → Well-being	**0.136**	**2.478**	**0.013**	**(0.028, 0.246)**
Emotional recovery → Well-being	0.043	0.664	0.507	(−0.087, 0.167)
BQ →Environmental improvement → Wellbeing	**0.180**	**5.085**	**<0.001**	**(0.114, 0.254)**
BA → Recreational activity → Wellbeing	**0.050**	**2.139**	**0.033**	**(0.010, 0.102)**
BM →Environmental improvement → Wellbeing	**−0.098**	**2.314**	**0.021**	**(−0.184, −0.021)**
FB → Well-being	0.059	1.055	0.291	(−0.052, 0.169)
FG → Well-being	0.050	1.044	0.296	(−0.047, 0.140)
TO → Well-being	−0.030	0.666	0.505	(−0.121, 0.058)

The bold values indicate *P* < 0.05.

Regarding the relationship between the subjective experience and subjective perception of blue spaces in urban parks, the results are as follows: First, for social interaction (SI), three dimensions of subjective experience, including attraction (BA, *β* = 0.194, *p* < 0.01), facility (BF, *β* = 0.370, *p* < 0.001), and management (BM, *β* = 0.288, *p* < 0.001), had significant and positive effects. In contrast, quality (BQ) showed a significant negative association with SI (*β* = −0.163, *p* < 0.05). Second, for environmental improvement (EI), BQ had a significant and positive effect (*β* = 0.447, *p* < 0.001), while BM was significantly and negatively associated (*β* = −0.242, *p* < 0.05). The effects of BA and BF were negative but statistically insignificant. Third, in the case of recreational activity (RA), both BA (*β* = 0.368, *p* < 0.001) and BF (*β* = 0.181, *p* < 0.05) showed significant and positive relationships, whereas BQ and BM had weak and non-significant effects. Fourth, for emotional recovery (ER), BA (*β* = 0.209, *p* < 0.01), BF (*β* = 0.240, *p* < 0.01), and BM (*β* = 0.284, *p* < 0.001) all demonstrated significant and positive effects, while the relationship with BQ remained weak and non-significant.

Among the indirect effects, only three pathways exhibited statistically significant associations with well-being. First, the subjective experience of quality (BQ) positively influenced well-being through the subjective perception of environmental improvement (EI, *β* = 0.180, *p* < 0.001). Second, the subjective experience of attractiveness (BA) positively affected well-being via the subjective perception of recreational activity (RA, *β* = 0.050, *p* = 0.033). Third, the subjective experience of management (BM) showed a significant negative indirect effect on well-being, also mediated by environmental improvement (EI, *β* = −0.098, *p* = 0.021).

**Figure 3:**
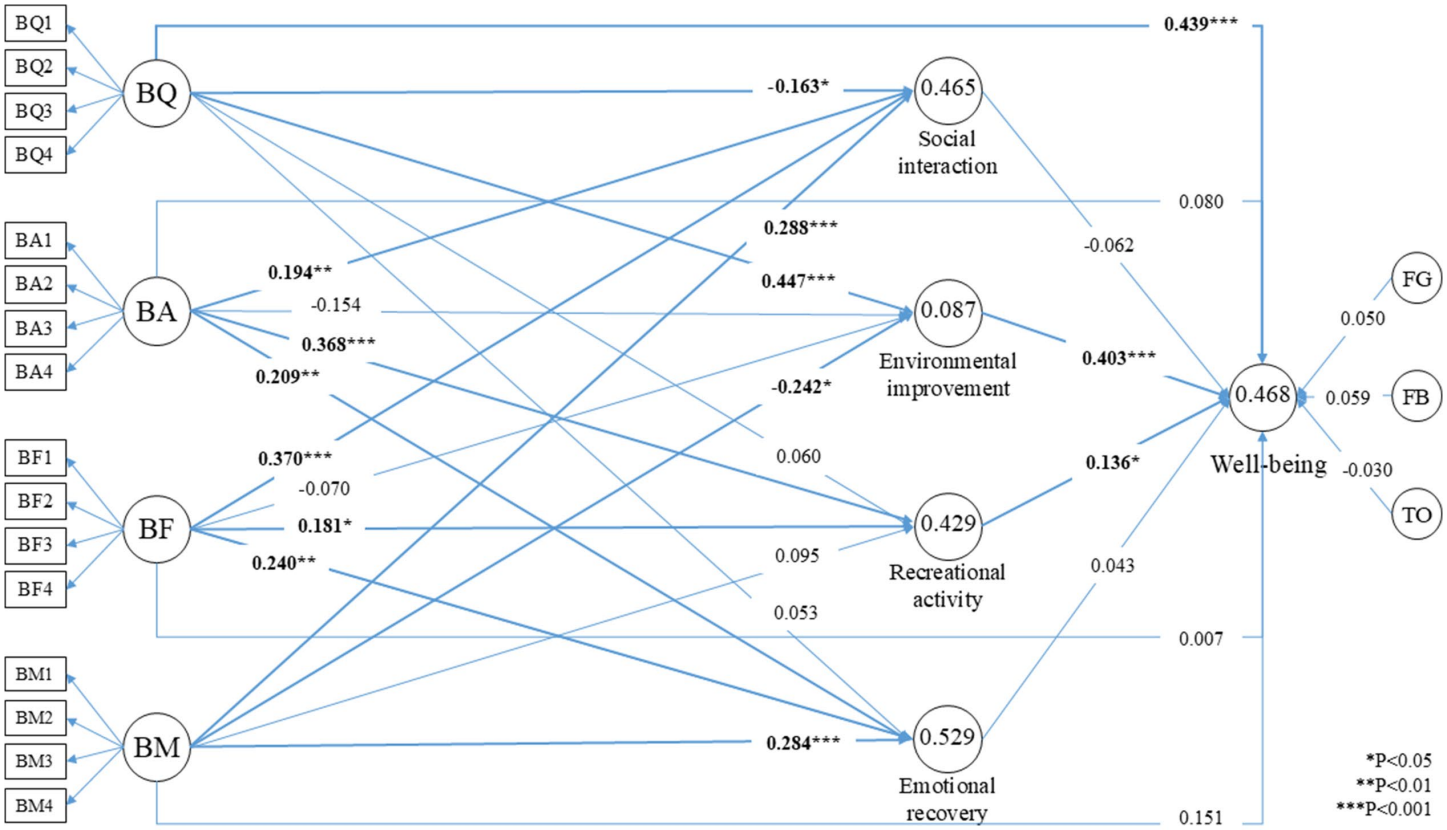
The results of PLS-SEM.

Figure 3 presents the path coefficients and corresponding *p*-values for the relationships between respondents’ subjective experience and perception of blue space in selected parks and their individual well-being. Table 6 summarizes the total effects and statistically significant indirect effects identified through the mediation analysis. Overall, the model demonstrated a good level of explanatory power, with a coefficient of determination (R^2^) of 0.468, indicating that the subjective experience variables account for a substantial proportion of the variance in individual well-being. Furthermore, the standardized root mean square residual (SRMR) was 0.070, which is below the commonly accepted threshold of 0.08, suggesting that the model exhibits an acceptable level of goodness of fit (11, 31, 49).

## 4 Discussion

This study focuses on the relationship between blue space and health, addressing two core research objectives: first, to examine the association between the subjective experience of urban-inland blue space in public parks and individual well-being; and second, to test whether subjective perception mediates this relationship. Based on empirical evidence derived from the PLS-SEM model, the findings demonstrate a significant relationship between the subjective experience of urban-inland blue space in public parks and residents’ well-being, with certain dimensions exerting indirect effects through pathways of subjective perception. The following discussion elaborates on the relationships among the variables, the implications of key mediating pathways, and the theoretical and practical contributions of the findings. Limitations and directions for future research are also addressed.

The results indicate that among the dimensions of subjective experience related to urban-inland blue spaces in public parks, only quality (BQ) had a significant positive direct effect on individual well-being (*β* = 0.439, *p* < 0.001). Based on the subcomponents of quality, this finding suggests that urban residents are more likely to evaluate the benefits of these blue spaces in terms of their tangible qualities—such as water color, waterfront, and the surrounding environment (see Section 2.3). This strong positive association further supports the notion that subjective experiences of blue space may have a more direct impact on individuals’ psychological states (18, 31). In contrast, other dimensions of subjective experience, namely attraction (BA), facility (BF), and management (BM), did not show significant direct effects on participants’ well-being. One possible explanation is that the survey emphasized participants’ subjective experience of urban-inland blue spaces in public parks, and general respondents may be less sensitive or accurate in evaluating elements beyond the water features themselves (31). It may also reflect that, compared to quality, other dimensions of experiences are more dependent on mediating processes involving personal perceptual interpretation.

Within the dimension of subjective perception, both environmental improvement (EI) and recreational activity (RA) played significant and positive mediating roles in the relationship between subjective experience and well-being. Among them, EI demonstrated the strongest effect (*β* = 0.403, *p* < 0.001). This finding differs from some existing studies (13), and a possible explanation lies in the differences in data collection methods. Some studies based on objective environmental measurements, such as air pollution or noise levels, suggest that small-scale blue spaces have limited capacity to improve environmental quality at the regional level (1, 30). However, our findings indicate that such spaces may still enhance individuals’ perception of environmental quality. A recent study supports this perspective: for example, an investigation on biodiversity in public green spaces and mental health found that perceived biodiversity was significantly associated with mental health through multiple pathways, while objectively measured biodiversity showed no such relationship (31). The discrepancy between subjective perception and objective measurement may account for the differences in their respective impacts on outcome variables, which warrants further investigation in future studies. Additionally, consistent with the findings of most previous studies (11, 18), the RA indicator was found to have a significant positive effect on individual well-being (*β* = 0.136, *p* < 0.05). This result suggests that some key pathways linking urban-inland blue space in public parks and individual health outcomes (24, 25), such as mitigation (harm reduction, e.g., EI in this study) and instoration (capacity building, e.g., RA in this study), can also be validated through subjectively perceived data.

Among the indirect effects, three key pathways demonstrated significant associations. First, quality indicators (BQ) was found to enhance well-being indirectly through environmental improvement (EI,*β* = 0.180, *p* < 0.001). This result is consistent with most existing evidence, which suggests that blue spaces with higher environmental quality can positively influence individual well-being (11, 18, 43). Specifically, the study examined participants subjective experiences of four aspects of urban-inland blue space quality in public parks (see Section 2.3, BQ1-BQ4), emphasizing the importance of optimizing spatial quality and enhancing residents’ experiences in urban-inland park blue spaces. Second, attraction indicators (BA) was shown to increase well-being through the promotion of recreational activity (RA, *β* = 0.050, *p* = 0.033), a finding supported by previous research (11, 37). It is noteworthy that the blue spaces examined in this study are integrated within public parks in urban-inland, and the primary activities undertaken by participants during their visits were most likely recreational (32, 36). The blue space in the park enhances visitors’ interest in visiting the park through its unique aesthetic appeal, thereby encouraging participants to engage in recreational activities that are beneficial to both physical and mental health. Third, management indicators (BM) exhibited a weak but statistically significant negative indirect effect on well-being through environmental improvement (EI, *β* = −0.098, *p* = 0.021). Given that the management indicators assessed in this study focused on artificial or human-centered management rather than ecological stewardship (see Section 2.3, BM1-BM4), this finding may reflect that inappropriate or overly manicured management practices could undermine residents’ trust in the natural environment’s restorative and ecological benefits (50). Moreover, such practices might lead residents to perceive these spaces as less natural and more anthropogenic, further diminishing their perceived psychological and ecological value.

This study further explored the pathways through which different dimensions of subjective experience influence subjective perception, revealing significant variability in how these dimensions shape individual cognition. First, quality indicators (BQ) demonstrated a significant negative effect on the social interaction (SI, *β* = −0.163, *p* < 0.05) dimension of subjective perception, which contrasts with some previous findings (51, 52). One possible explanation is that high-quality blue spaces in urban parks tend to possess characteristics such as solitude and tranquility, encouraging activities like solitary meditation or observation rather than social interaction. These assumptions warrant further in-depth investigation to validate their underlying mechanisms. In contrast, BQ showed a significant positive effect on environmental improvement (EI, *β* = 0.447, *p* < 0.001), indicating that individuals are more likely to associate high-quality park’s blue spaces with ecological enhancement, fostering stronger environmental awareness (25). However, the effects of BQ on recreational activity (RA) and emotional restoration (ER) were not statistically significant.

Second, attraction indicators (BA) demonstrated significant positive effects on several dimensions of subjective perception, including social interaction (SI, *β* = 0.194, *p* < 0.01), recreational activity (RA, *β* = 0.368, *p* < 0.001), and emotional restoration (ER, *β* = 0.209, *p* < 0.01). These findings suggest that highly attractive blue spaces may enhance visitor’s sense of engagement and enjoyment, thereby fostering increased social contact and positive emotional experiences (18, 44). However, the effect of BA on environmental improvement (EI) was not statistically significant, which may be due to participants perceiving attractiveness primarily in terms of aesthetic and design features, rather than ecological quality.

Third, facilities indicators (BF) also exerted multiple significant positive effects on subjective perception. BF was positively associated with social interaction (SI, *β* = 0.370, *p* < 0.001), recreational activity (RA, *β* = 0.181, *p* < 0.05), and emotional restoration (ER, *β* = 0.240, *p* < 0.01). These results support the notion that well-designed and adequate facilities provide residents with opportunities for staying, socializing, and engaging in activities, thereby enhancing both recreational experience and emotional regulation (11, 18). However, the effect of BF on environmental improvement (EI) was not significant, possibly because residents do not readily associate man-made infrastructure with improvements in ecological conditions.

Fourth, management indicators (BM) showed significant positive effects on social interaction (SI, *β* = 0.288, *p* < 0.001) and emotional restoration (ER, *β* = 0.284, *p* < 0.001), indicating that effective management contributes to a more comfortable and secure environment, enhancing residents’ feelings of safety and controllability (42), which in turn promotes social connections and emotional recovery. In contrast, BM had a significant negative effect on environmental improvement (EI, *β* = −0.242, *p* < 0.05). This may be explained by the perception that overly intensive management interventions can reduce the naturalness of the space (50), thereby weakening the perceived ecological benefits. This highlights the need to balance manageability and naturalness in the design and maintenance of urban-inland blue spaces within public parks.

The control variables in the research model, including frequency of green space use (FG), frequency of blue space use (FB), and time spent on outdoor (TO), did not show statistically significant associations with well-being. This suggests that their direct effects on individual well-being were relatively weak within the current sample. This finding contrasts with some previous studies that reported positive associations between objective exposure indicators, such as frequency and duration of contact with natural spaces, and mental health (4, 28, 43). However, an increasing number of recent studies have emphasized that the quality of subjective experiences may be a stronger predictor of health benefits than the quantity of exposure (53) and our findings provide support for this emerging perspective. Moreover, this study focused on the subjective experience and perceived pathways of blue space within urban parks and incorporated multiple perception-related variables as mediators in the model. These mediators may have partially absorbed the effects of exposure frequency. Therefore, from a statistical standpoint, the direct paths from FG, FB, and TO variables to individual well-being might have been overshadowed by more explanatory subjective variables.

This study explores the pathway linking subjective experience and perception, as well as individual well-being, aiming to elucidate the mechanisms between urban-inland blue spaces in public parks and residents’ well-being. Focusing on inland cities, an area that has received relatively limited attention in prior research, this study examines five representatives public parks as case studies. It provides a comparative perspective on residents’ experiences and perceptions of blue space in urban parks, offering valuable empirical data and insights for future research on inland urban blue spaces. The findings suggest that urban planners and policymakers should place greater emphasis on enhancing the quality, attraction, facility, and management of blue spaces in public parks. Improving residents’ subjective experiences can strengthen their perceived benefits in terms of social interaction, environmental improvement, recreation activity, and emotional restoration, thereby enhancing the ecological service value of urban living environments and contributing to public health promotion.

The study has several limitations that warrant further improvement in future research. First, the use of cross-sectional data limits the ability to infer causality. Future studies could adopt longitudinal or experimental designs to more systematically examine the causal mechanisms linking subjective experiences of blue space to well-being. Second, the reliance on self-reported measures may introduce subjective bias. Subsequent research could incorporate multi-source data, such as behavioral tracking, objective environmental assessments, or physiological indicators, to enable cross-validation. Third, due to the limited sample size, this study was unable to conduct in-depth multi-group modeling analyses of different types of blue spaces across parks. While visit-related variables were included as covariates, individual-level characteristics were not incorporated, considering model complexity and sample limitations. Future research could further explore the differential effects of various types of urban-inland blue spaces and examine the potential influence of individual characteristics. Fourth, data collection was mainly conducted during seasons favorable for outdoor activities, which may have led to an overestimation of the positive effects of blue space. Residents’ needs, frequency of use, and perceived experiences of blue space may vary significantly across different seasons. Therefore, future studies are encouraged to conduct comparative analyses through cross-seasonal or year-round surveys.

## 5 Conclusion

Guided by established theoretical frameworks and methodologies, this study employed PLS-SEM approach to examine the relationship between the perceived experience of blue space in urban parks and individual well-being, as well as the potential mediating pathways involved. Participants reported their subjective experiences related to the quality, attraction, facility, and management of blue spaces during park visits. To investigate potential mediators, the model incorporated four indicators of subjective perception: social interaction, environmental improvement, recreational activity, and emotional recovery. Additionally, the frequency of visits to green and blue spaces, as well as duration of outdoor activity, were included as control variables to account for variability in individuals’ well-being outcomes.

The results revealed a significant association between residents’ subjective experience of blue space in urban parks and their well-being in inland urban contexts. Moreover, this relationship was partially mediated by subjective perception, highlighting its underlying psychological mechanisms. In particular, the two perception dimensions of environmental improvement and recreational activity play crucial roles in the pathway, while quality is the only experience indicator that directly influences individual well-being. This implies that enhancing the visual quality and recreational value of urban-inland blue spaces in public parks is a key entry point for improving well-being outcomes. Future research should adopt longitudinal or experimental designs to verify causal relationships, integrate objective indicators to reduce bias, compare the effects of different types of blue spaces and individual differences, and consider seasonal factors in cross-period surveys. The study results underscore the crucial role of subjective experience and perception of urban parks’ blue spaces in shaping individual well-being. It emphasizes the need to consider not only the physical availability of blue spaces, but also how people experience and perceive them, in order to maximize their well-being benefits. The findings offer valuable implications for design and planning of blue space in urban park, suggesting that enhancing the experiential and perceptional qualities of blue spaces can generate additional social and health benefits, thereby contributing to a more balanced relationship between urban development and nature. In conclusion, this study enriches the growing body of evidence linking blue space and public health, particularly in the context of inland cities, where blue space is often limited but can be strategically optimized for maximum benefit.

## Data Availability

The original contributions presented in the study are included in the article/Supplementary material, further inquiries can be directed to the corresponding author/s.
