# Supplementary material for: Subjective experience and perception of urban-inland blue spaces in urban parks and individual well-being: evidence from Xi’an, China
Source: Front Public Health. 2025 Oct 30;13:1621437. doi: 10.3389/fpubh.2025.1621437 (PMC12613235; doi:10.3389/fpubh.2025.1621437)
Supplement: Supplementary file 1 [file Data_Sheet_1.pdf]

Supplementary Material

Table S1. Basic information about the selected parks.

Park name	Brief description of selected park
**Xingqing Palace Park (XQ)**	Located in the central area, XQ is an urban park built on a historical site. It features a large artificial lake (Xingqing Lake), with arched bridges, waterfront platforms, and a lakeside walking trail. The park is well-vegetated, offers high water visibility, and serves as a primary location for residents' daily leisure and physical activities.
**Xi'an Hancheng Lake (HC)**	Situated in the northern area, HC is a large linear waterfront green space characterized by interconnected artificial lakes and urban watercourses. It includes an extensive waterside trail system and a variety of waterfront facilities. With a high degree of ecological restoration, it serves as a typical water-dominated urban green space.
**Peach Blossom Tan Park (PB)**	Located in the eastern suburbs, PB is a community-oriented park containing small- to medium-sized artificial lakes and natural wetland streams. It provides some ecological functions and is equipped with fitness facilities and children's play areas, serving as an important public space for nearby residents.
**Xi'an ChanBa National Wetland Park (CB)**	Located in the northeastern area, CB is an ecological park centered on wetland ecosystems. It contains multiple natural river confluences and wetland shoals, with a high proportion of blue space. The park is rich in vegetation, frequently visited by wild waterfowl, and holds significant ecological and environmental education value.
**Qujiang Pool Relic Park (QJ)**	Situated in a historical and cultural area, QJ was developed based on the site of a Tang Dynasty royal water landscape. It features artificial ponds and lakeside historic architecture. Combining historical heritage with modern landscape design, the park integrates cultural display with recreational use, serving as a culturally themed urban water park.

Table S2. Measurement items of covariates.

Item	Question description	Response options
**FG**	How many days did you visit green spaces in the past week?	0 days, 1-2 days, 3-4 days, 5-6 days, every day
**FB**	How many days did you visit blue spaces in the past week?	0 days, 1-2 days, 3-4 days, 5-6 days, every day
**TO**	During the past week, how long did you spend outdoors on average each day?	less than 0.5 hours, 0.5 to 1 hour, 1 to 3 hours, 3-5 hours, more than 5 hours.

Table S3. One-way ANOVA results for well-being across parks.

Park name	N	Mean	SD	F	P
**XQ**	84	82.62	10.40	7.667	<0.001
**HC**	72	82.11	8.38		
**PB**	95	77.68	11.71		
**CB**	97	75.34	19.33		
**QJ**	81	74.12	8.00		

XQ=Xingqing Palace Park, HC=Xi'an Hancheng Lake, PB=Peach Blossom Tan Park, CB=Xi'an ChanBa National Wetland Park, QJ=Qujiang Pool Relic Park.

Table S4. Bonferroni-adjusted multiple comparisons of well-being among parks.

Park name	XQ	HC	PB	CB	QJ
**XQ**	1	1.000	0.095	0.001	<0.001
**HC**	1.000	1	0.256	0.006	0.001
**PB**	0.095	0.256	1	1.000	0.634
**CB**	0.001	0.006	1.000	1	1.000
**QJ**	<0.001	0.001	0.634	1.000	1

XQ=Xingqing Palace Park, HC=Xi'an Hancheng Lake, PB=Peach Blossom Tan Park, CB=Xi'an ChanBa National Wetland Park, QJ=Qujiang Pool Relic Park.
